# Nivolumab provides improved effectiveness and safety compared with docetaxel as a second‐line treatment for advanced non‐small cell lung cancer: A systematic review and meta‐analysis

**DOI:** 10.1002/cam4.1966

**Published:** 2019-01-09

**Authors:** Zheng Xu, Fengming Yi, Dongliang Yu, Jianjun Xu, Yiping Wei, Wenxiong Zhang

**Affiliations:** ^1^ Department of Cardio‐Thoracic Surgery The Second Affiliated Hospital of Nanchang University Nanchang China; ^2^ Department of Oncology The Second Affiliated Hospital of Nanchang University Nanchang China; ^3^ Department of Thoracic Surgery The Second Affiliated Hospital of Nanchang University Nanchang China

**Keywords:** chemotherapy, docetaxel, meta‐analysis, nivolumab, non‐small cell lung cancer

## Abstract

**Background:**

As an inhibitor of programmed death‐1 (PD‐1) protein, nivolumab has been shown to be effective in various cancers. We thus conducted this meta‐analysis to compare the relative efficacy of nivolumab vs docetaxel‐based chemotherapy as a second‐line treatment for previously treated advanced non‐small cell lung cancer (NSCLC).

**Methods:**

Relevant studies were identified through searches of databases and conference proceedings. Progression‐free survival (PFS), overall survival (OS), drug responses, and adverse effects (AEs) were assessed as the primary endpoints.

**Results:**

After screening, we included six studies (949 patients) in the final analysis. Nivolumab showed better efficacy in terms of the PFS (hazard ratios [HR]: 0.70, *P* = 0.03), OS (HR: 0.70, *P* < 0.00001), objective response rate (ORR) (risk ratios [RR]: 1.73, *P* = 0.0008), total AEs (RR: 0.77, *P* = 0.006), and grade 3‐5 AEs (RR: 0.18, *P* < 0.00001) than docetaxel. The anti‐tumor efficacy of nivolumab for NSCLC in terms of both PFS and OS was positively correlated with the level of PD‐L1 expression. In the nivolumab treatment arm, the 10 most‐reported AEs were fatigue (15.7%), nausea (10.8%), decreased appetite (10.3%), asthenia (9.8%), diarrhea (7.5%), rash (7.5%), arthralgia (5.4%), vomiting (4.4%), constipation (3.5%), and pyrexia (3.3%).

**Conclusions:**

For advanced NSCLC, nivolumab is a better therapy in terms of both anti‐tumor efficacy and safety than docetaxel‐based chemotherapy. More high‐quality randomized controlled trials are needed to confirm these results.

## 1 INTRODUCTION

Lung cancer remains the primary cause of cancer‐related deaths worldwide.^1^ Despite significant therapeutic advances in targeted therapies, antiangiogenics, and chemotherapeutic drugs, their curative effects remain dismal.^2^, ^3^ In recent years, research studies investigating immune checkpoint inhibitors (ICIs) that target the PD‐1/PD‐L1 (programmed death‐1/programmed death ligand‐1) pathway have made significant breakthroughs, resulting in significantly longer survival rates than those obtained with the standard of care approved by the US Food and Drug Administration (FDA) for non‐small cell lung cancer (NSCLC).^4^, ^5^, ^6^ Compared with other PD‐1 inhibitors (pembrolizumab and atezolizumab), treatment with nivolumab results in similar survival outcomes and better safety outcomes.^7^

Docetaxel‐based chemotherapy is considered the standard second‐line treatment for patients who relapsed after first‐line platinum‐based chemotherapy or targeted therapy, but its curative effect is less satisfactory and accompaniedby substantial toxicity.^8^ A phase III RCT conducted by Borghaei et al^9^ revealed that treatment with nivolumab resulted in longer overall survival than docetaxel for advanced nonsquamous NSCLC after platinum‐based chemotherapy. In another phase III RCT, nivolumab achieved significantly better overall survival (OS), response rate, and progression‐free survival (PFS) than docetaxel for advanced squamous‐cell NSCLC after platinum‐based chemotherapy,^10^ and 2‐ and 3‐year updates to these data continued to yield encouraging results.^11^, ^12^ Thus, these recent studies have challenged the paradigm of treatment of patients with NSCLC who relapsed after previous treatment.

With the goal of identifying a better second‐line therapeutic regimen for patients with advanced NSCLC, we conducted a meta‐analysis to compare the anti‐tumor efficacy and adverse effects (AEs) between nivolumab and docetaxel.

## 2 MATERIALS AND METHODS

We conducted this meta‐analysis according to the Preferred Reporting Items for Systematic Review and Meta‐Analysis guidelines (PRISMA) (Table S1).

### 2.1 Search strategy

PubMed, EMBASE, Ovid MEDLINE, Scopus, Web of Science, Cochrane Library, ScienceDirect, Ovid MEDLINE, and Google Scholar were rigorously searched for prospective cohort studies from their inception to 5 June 2018. The following keywords were used: “nivolumab,” “docetaxel,” and “lung cancer.” The references of the retrieved articles were also searched for further eligible articles.

### 2.2 Selection criteria

The inclusion criteria were the following:

1. Population: patients with stage III/IV NSCLC.
2. Intervention and comparison: nivolumab was compared with docetaxel.
3. Outcomes: OS, PFS, objective response rate (ORR), complete response (CR), partial response (PR), stable disease (SD), progressive disease (PD), disease control rate (DCR), and AEs.
4. Study design: high‐quality cohort studies and randomized controlled trials (RCTs).

We excluded reviews without original data, meta‐analyses, animal‐based studies, abstracts only, and studies with duplicated data.

### 2.3 Data extraction

Two reviewers independently extracted and summarized the following data: first author, publication year, region, study design, number of participants, tumor histology, clinical stage, EGFR status, anti‐tumor efficacy indices (PFS, OS, ORR, DCR, CR, PR, SD, and PD), and number of AEs (total AEs and grade 3‐5 AEs). A third investigator resolved any disagreements.

### 2.4 Outcome assessments

PFS, OS, ORR, and AEs were assessed as the main outcomes, and we also analyzed the CR, PR, SD, and PD rates to perform a component analysis of the ORR. In the subgroup analysis according to PD‐L1 expression (1%, 5%, 10%, and 50%), the anti‐tumor efficacy (OS and PFS) was compared between two groups. Subgroup analyses of PFS, OS, and ORR were also conducted to determine whether the results changed according to ECOG, histology, and study design. To analyze the treatment effect over time, we compared the one‐, two‐, and three‐year outcomes of two RCTs (Checkmate 017 and Checkmate 057).

### 2.5 Quality assessment

We used the Cochrane Risk of Bias Tool to perform a quality assessment of each trial. The evaluation indicators included the randomization sequence generation, allocation concealment, blinding, incomplete outcome data, and selective reporting. The quality of each study was categorized as high, low, or unclear.^13^

### 2.6 Statistical analysis

The meta‐analysis was performed using Review Manager 5.3 (Te Nordic Cochrane Centre, Te Cochrane Collaboration, Copenhagen, Denmark) and STATA 12.0 (Stata Corp LP, College Station, TX, USA). Hazard ratios (HR) were used to analyze the PFS and OS (HR > 1 favors the docetaxel group; HR < 1 favors the nivolumab group), and pooled risk ratios (RR) were used to analyze the ORR, CR, PR, SD, PD, DCR, and AEs. The chi‐square test and *I* ^2^ statistic were used for the evaluation of heterogeneity. If *I* ^2 ^< 50% or *P* > 0.1, which reflected low heterogeneity, the fixed‐effects model was used; otherwise, the random‐effects model was used. *P* < 0.05 was considered to indicate statistical significance. Publication bias was explored using Begg's rank correlation and Egger's linear regression tests.

## 3 RESULTS

### 3.1 Search results and study quality assessment

**Figure 1:**
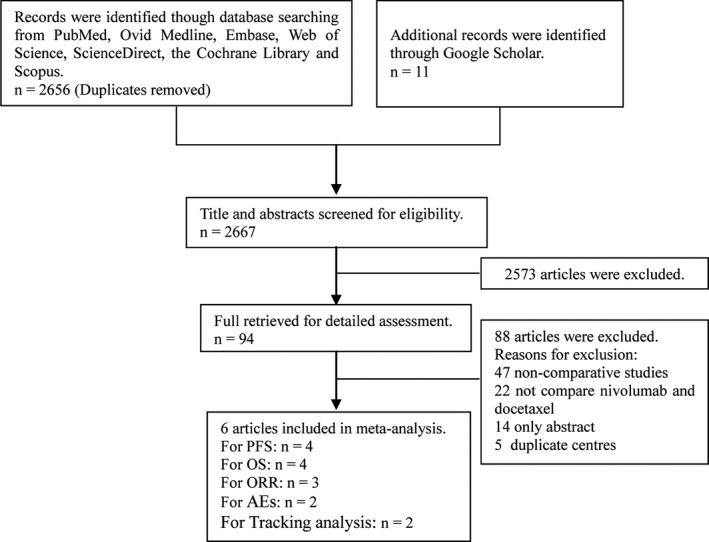
Flow chart of study selection

We initially identified 2667 potentially eligible studies, and after screening, six studies involving 949 patients (469 patients in the nivolumab group, and 480 patients in the docetaxel group) were included in the final analysis (Figure 1).^9^, ^10^, ^11^, ^12^, ^14^, ^15^ Of the six studies, four were RCTs (studies 11 and 12 were the two‐year and three‐year outcomes of studies 9 and 10), and two were retrospective studies. According to the Cochrane Risk of Bias Tool, all the included studies were of high quality (Figure S1). Table 1 summarizes the baseline characteristics and main evaluation indices of the included studies.

**Table 1:** Characteristics of included studies

Study		Country	Treatment arms	Treatment line	Previous treatment	Patients (n)	Median age (y)	ECOG status	Follow‐up duration, (mo)	Pathology	Stage	Design
2015	Borghaei^9^	USA	Nivolumab, 3 mg/kg, q2w	2 or 3	Platinum‐based chemotherapy	292	61	0 or 1	13.2	Squamous NSCLC	IIIb, IV	RCT
			Docetaxel, 75 mg/m^2^, q3w			290	64					
2015	Brahmer^10^	USA	Nivolumab, 3 mg/kg, q2w	2	Platinum‐based chemotherapy or TKI	135	62	0 or 1	11	Nonsquamous NSCLC	IIIb, IV	RCT
			Docetaxel, 75 mg/m^2^, q3w			137	64					
2017	Horn^11^	USA	Nivolumab, 3 mg/kg, q2w	2 or 3	Platinum‐based chemotherapy or TKI	427	61	0 or 1	24.2	NSCLC	IIIb, IV	RCT
			Docetaxel, 75 mg/m^2^, q3w			427	64					
2018	Vokes^12^	USA	Nivolumab, 3 mg/kg, q2w	2 or 3	Platinum‐based chemotherapy or TKI	427	61	0 or 1	40.3	NSCLC	IIIb, IV	RCT
			Docetaxel, 75 mg/m^2^, q3w			427	64					
2017	Pablo^14^	Spain	Nivolumab, 3 mg/kg, q2w	2	Platinum‐based chemotherapy	14	65.5	0 or 1(11), ≥2(3)	3.9	NSCLC	III, IV	RS
			Docetaxel, 75 mg/m^2^, q3w			19	64.6	0 or 1(10), ≥2(9)				
2018	Russo^15^	Italy	Nivolumab, 3 mg/kg, q2w	2 or 3	Platinum‐based chemotherapy	28	69	N/A	N/A	NSCLC	III, IV	RS
			Docetaxel, 75 mg/m^2^, q3w			34	68					

ECOG, Eastern Cooperative Oncology Group; N/A, not available; NSCLC, non‐small cell lung cancer; RCT, randomized controlled trial; RS, retrospective study; TKI, tyrosine kinase inhibitor; USA, United States of America.

### 3.2 Anti‐tumor efficacy

**Figure 2:**
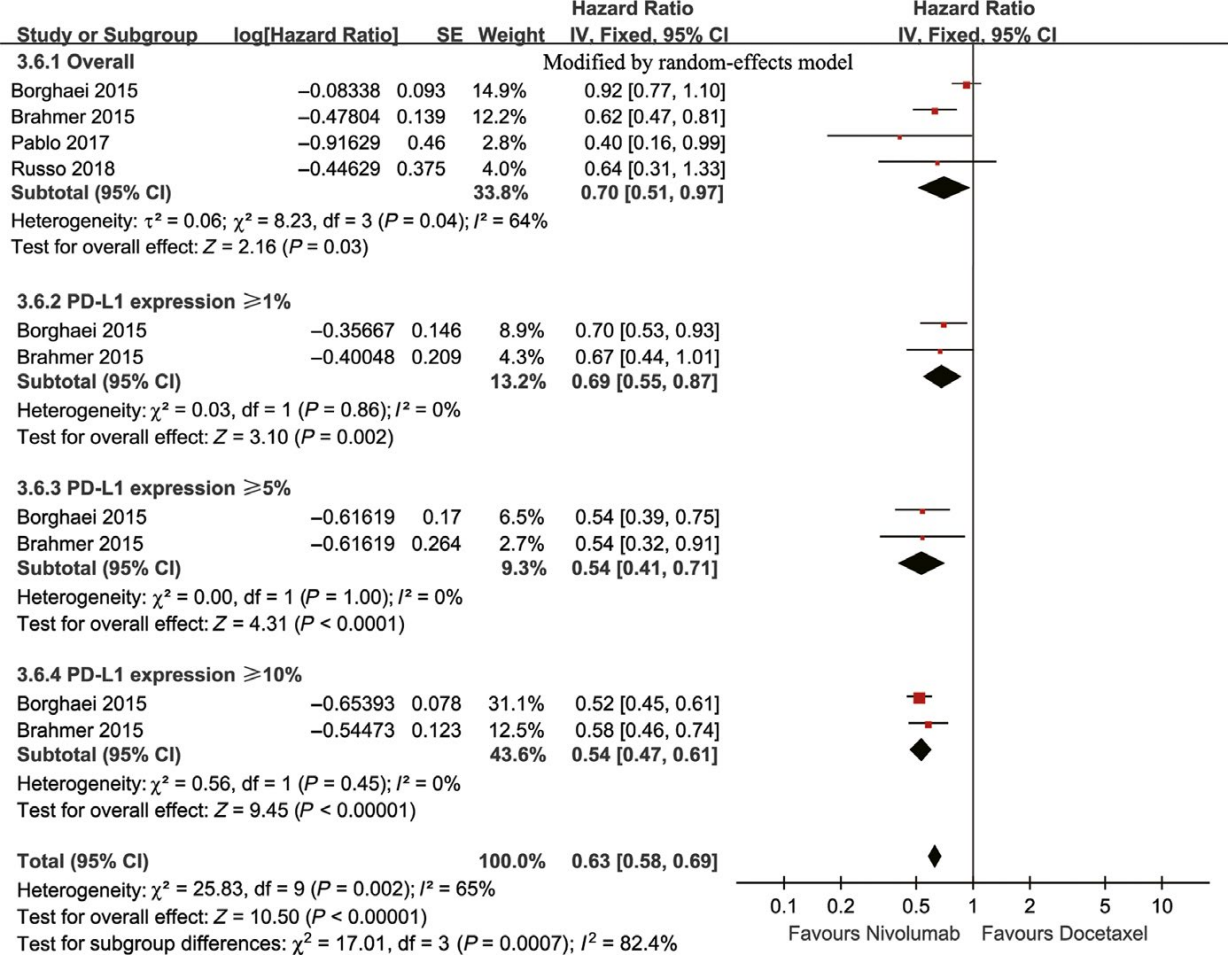
Forest plots of HR of PFS associated with nivolumab vs docetaxel (including subgroup analysis according to PD‐L1 expression)

Four studies compared PFS (heterogeneity: *P* = 0.04, *I* ^2^ = 64%), and the results revealed that nivolumab enhanced the PFS of the patients compared with docetaxel, regardless of the PD‐L1 expression level (HR: 0.70, 95% confidence interval [CI]: 0.51‐0.97, *P* = 0.03; Figure 2).

**Figure 3:**
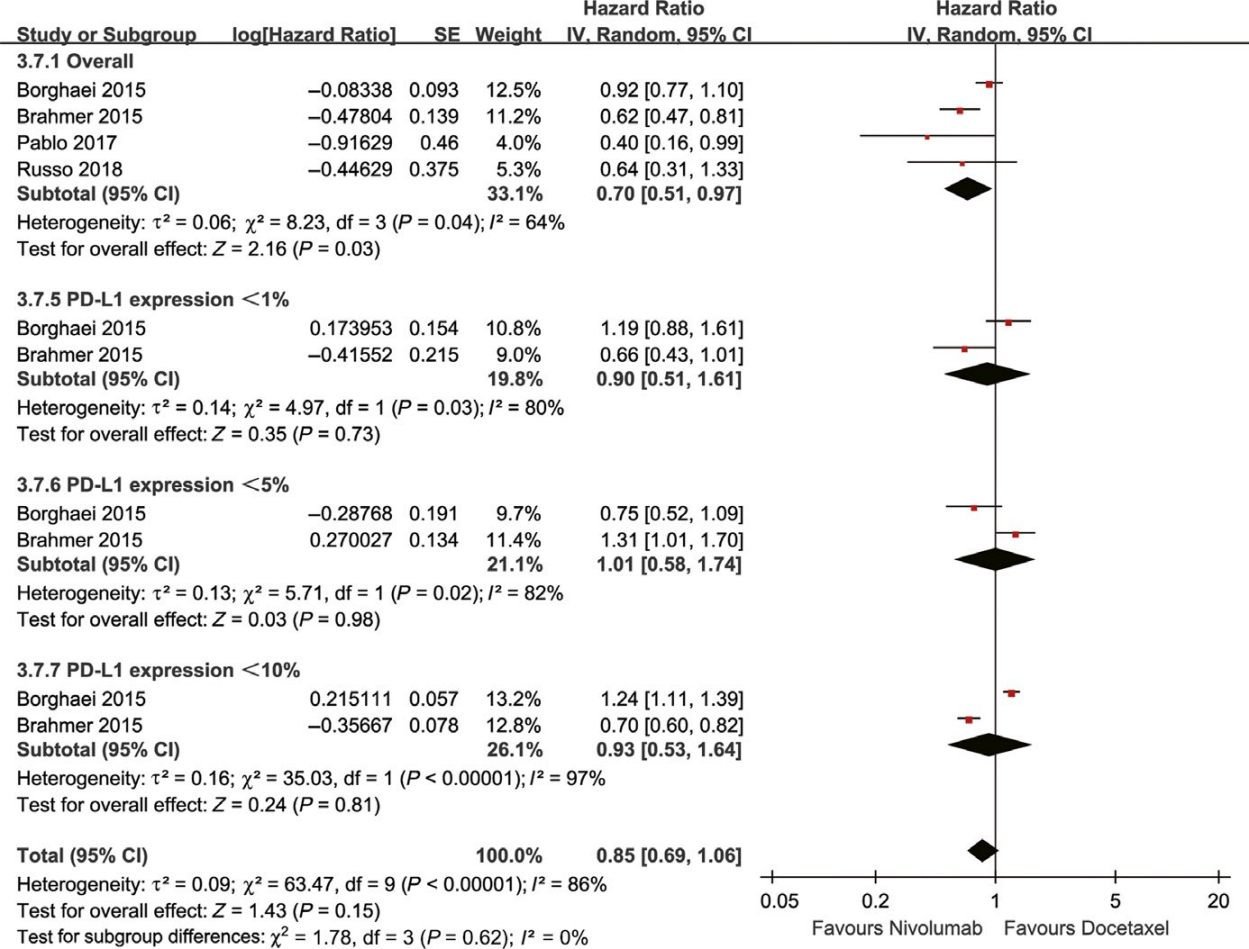
Forest plots of HR of OS associated with nivolumab vs docetaxel (including subgroup analysis according to PD‐L1 expression)

Three studies, which included four cohorts, compared OS (heterogeneity: *P* = 0.67, *I* ^2^ = 0%). The nivolumab group showed enhanced OS compared with the docetaxel group, regardless of the level of PD‐L1 expression (HR: 0.70, 95% CI: 0.60‐0.82, *P* < 0.00001; Figure 3).

**Figure 4:**
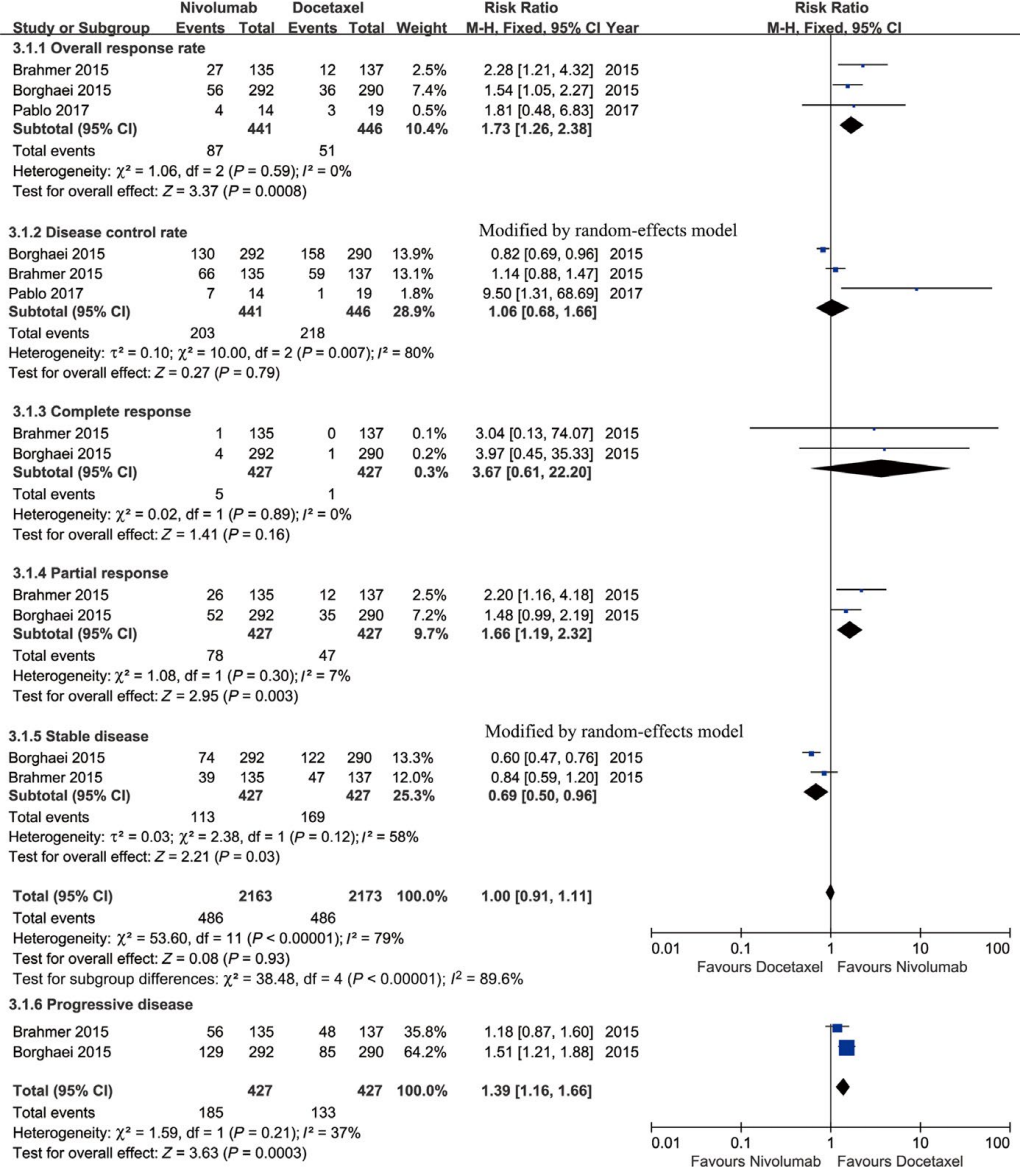
Forest plots of RR of drug responses associated with nivolumab vs docetaxel (including objective response rate, disease control rate, complete response, partial response, stable disease, and progressive disease)

Three studies compared ORR (heterogeneity: *P* = 0.59, *I* ^2^ = 0%), and the findings revealed that nivolumab enhanced the ORR compared with docetaxel (RR: 1.73, 95% CI: 1.28‐2.38, *P* = 0.0008; Figure 4). Further analysis indicated that the complete responses were comparable between the two groups (RR: 3.67, 95% CI: 0.61‐22.2, *P* = 0.16), and partial responses were more frequently observed in the nivolumab group (RR: 1.66, 95% CI: 1.19‐2.32, *P* = 0.003) (Figure 4). However, the nivolumab group included fewer patients with stable disease (RR: 0.69, 95% CI: 0.50‐0.96, *P* = 0.03) and more patients with progressive disease (RR: 1.39, 95% CI: 1.16‐1.66, *P* = 0.0003; Figure 4).

### 3.3 Toxicity

We performed a toxicity comparison based on the total number of grade 3‐5 AEs between the nivolumab and docetaxel groups and a subgroup analysis of the 10 most‐reported AEs.

**Figure 5:**
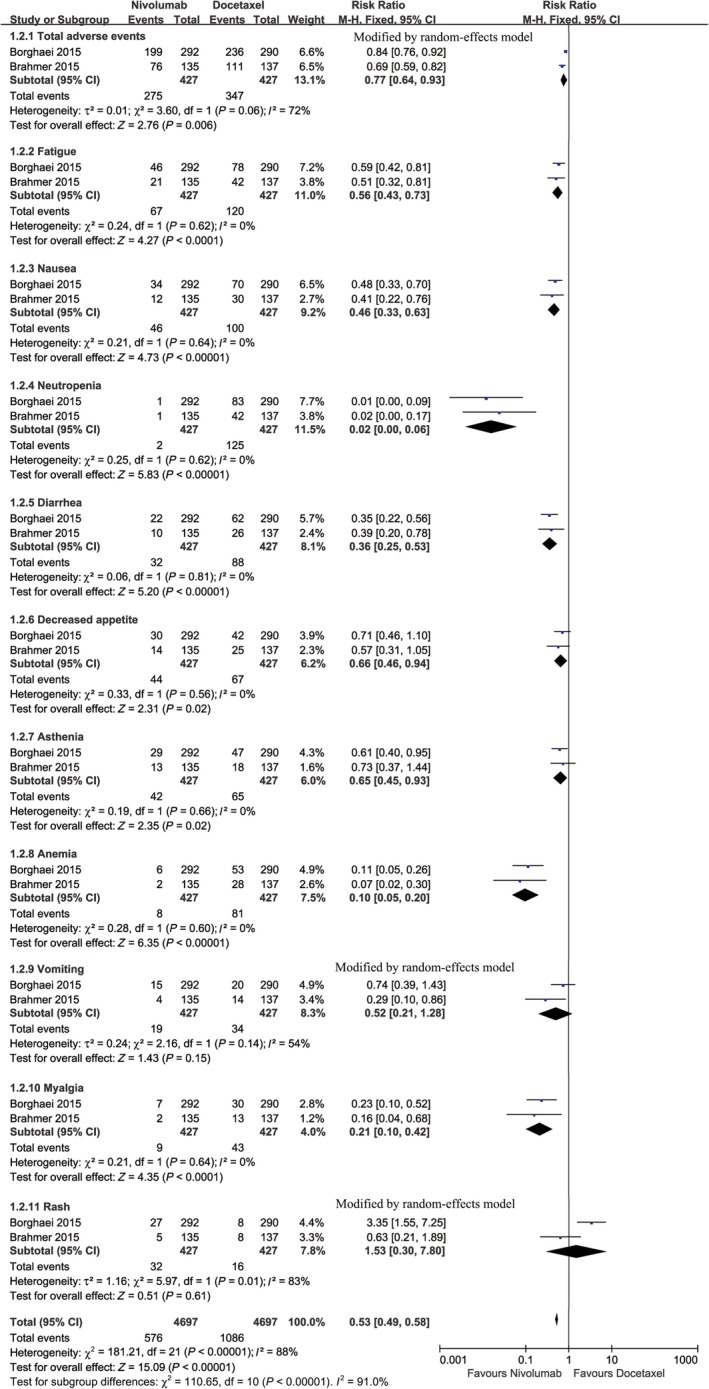
Forest plots of RR of all‐grade AEs associated with nivolumab vs docetaxel (including subgroup analysis of 10 most‐reported AEs according to the combination of both arms)

Two studies compared the total AEs (heterogeneity: *P* = 0.06, *I* ^2^ = 72%), and these found a higher number of AEs in the docetaxel group (RR: 0.77, 95% CI: 0.64‐0.93, *P* = 0.006; Figure 5). The 10 most‐reported AEs in the entire population were fatigue, nausea, neutropenia, diarrhea, decreased appetite, asthenia, anemia, vomiting, myalgia, and rash. No significant differences in risks of vomiting and rash were found between the two groups. Treatment with docetaxel induced a significantly higher risk of fatigue, nausea, neutropenia, diarrhea, decreased appetite, asthenia, anemia, and myalgia (Figure 5).

**Figure 6:**
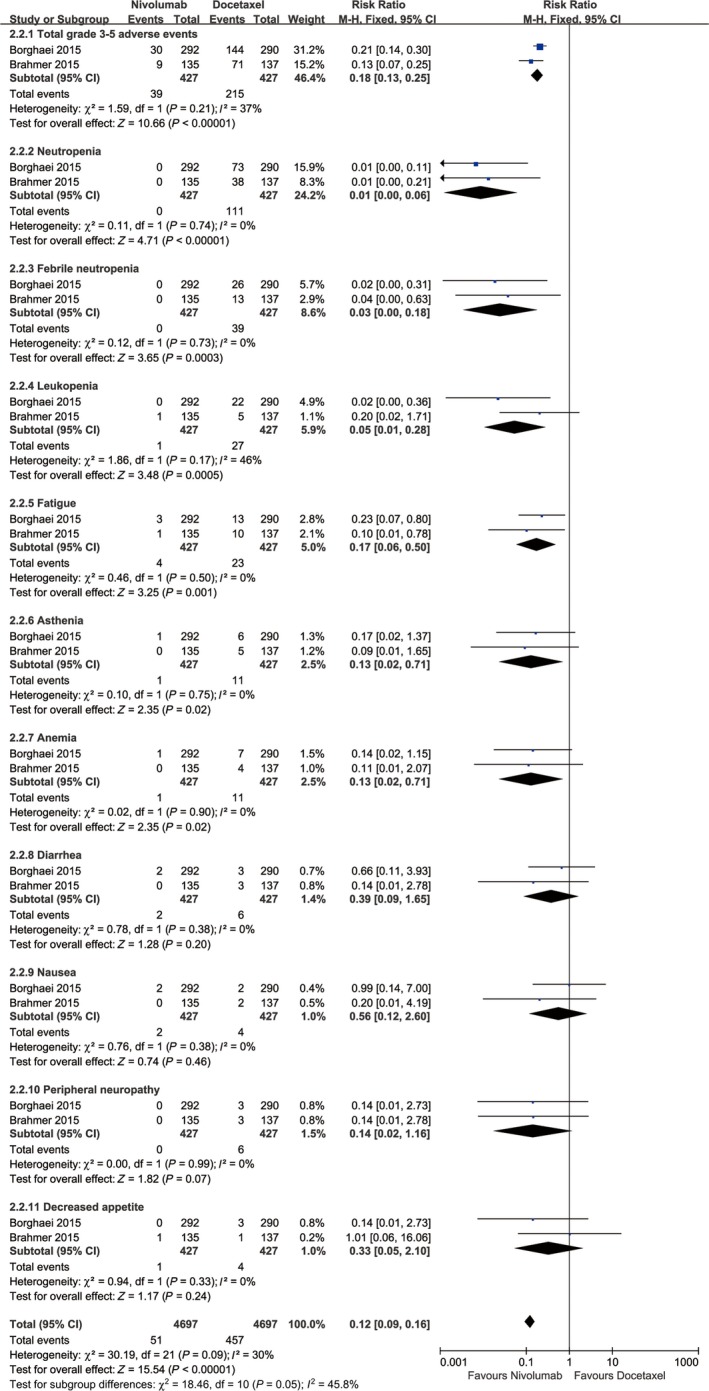
Forest plots of RR of grade 3‐5 AEs associated with nivolumab vs docetaxel (including subgroup analysis of 10 most‐reported grade 3‐5 AEs according to the combination of both arms)

Two studies compared grade 3‐5 AEs (heterogeneity: *P* = 0.21, *I* ^2^ = 37%) and found more AEs in the docetaxel group (RR: 0.18, 95% CI: 0.13‐0.25, *P* < 0.00001; Figure 6). In the entire population, the 10 most‐reported grade 3‐5 AEs were neutropenia, febrile neutropenia, leukopenia, fatigue, asthenia, anemia, diarrhea, nausea, peripheral neuropathy, and decreased appetite. No significant differences in risks of diarrhea, nausea, peripheral neuropathy, and decreased appetite were found between the two groups. The analysis of grade 3‐5 AEs revealed that docetaxel induced significantly higher rates of neutropenia, febrile neutropenia, leukopenia, fatigue, asthenia, and anemia (Figure 6).

In the nivolumab treatment arm, the 10 most‐reported AEs were fatigue (15.7%), nausea (10.8%), decreased appetite (10.3%), asthenia (9.8%), diarrhea (7.5%), rash (7.5%), arthralgia (5.4%), vomiting (4.4%), constipation (3.5%), and pyrexia (3.3%; Table 2).

**Table 2:** Top 10 adverse effects (all‐grade and grade 3‐5) in nivolumab group

Adverse effects	Total Grade		Grade 3‐5	
	Event/Total	%	Event/Total	%
Fatigue	67/427	15.69	4/427	0.94
Nausea	46/427	10.77	2/427	0.47
Decreased appetite	44/427	10.30	1/427	0.23
Asthenia	42/427	9.84	1/427	0.23
Diarrhea	32/427	7.49	2/427	0.47
Rash	32/427	7.49	1/427	0.23
Arthralgia	23/427	5.39	0/427	0.00
Vomiting	19/427	4.45	0/427	0.00
Constipation	15/427	3.51	0/427	0.00
Pyrexia	14/427	3.28	0/427	0.00

**Figure 7:**
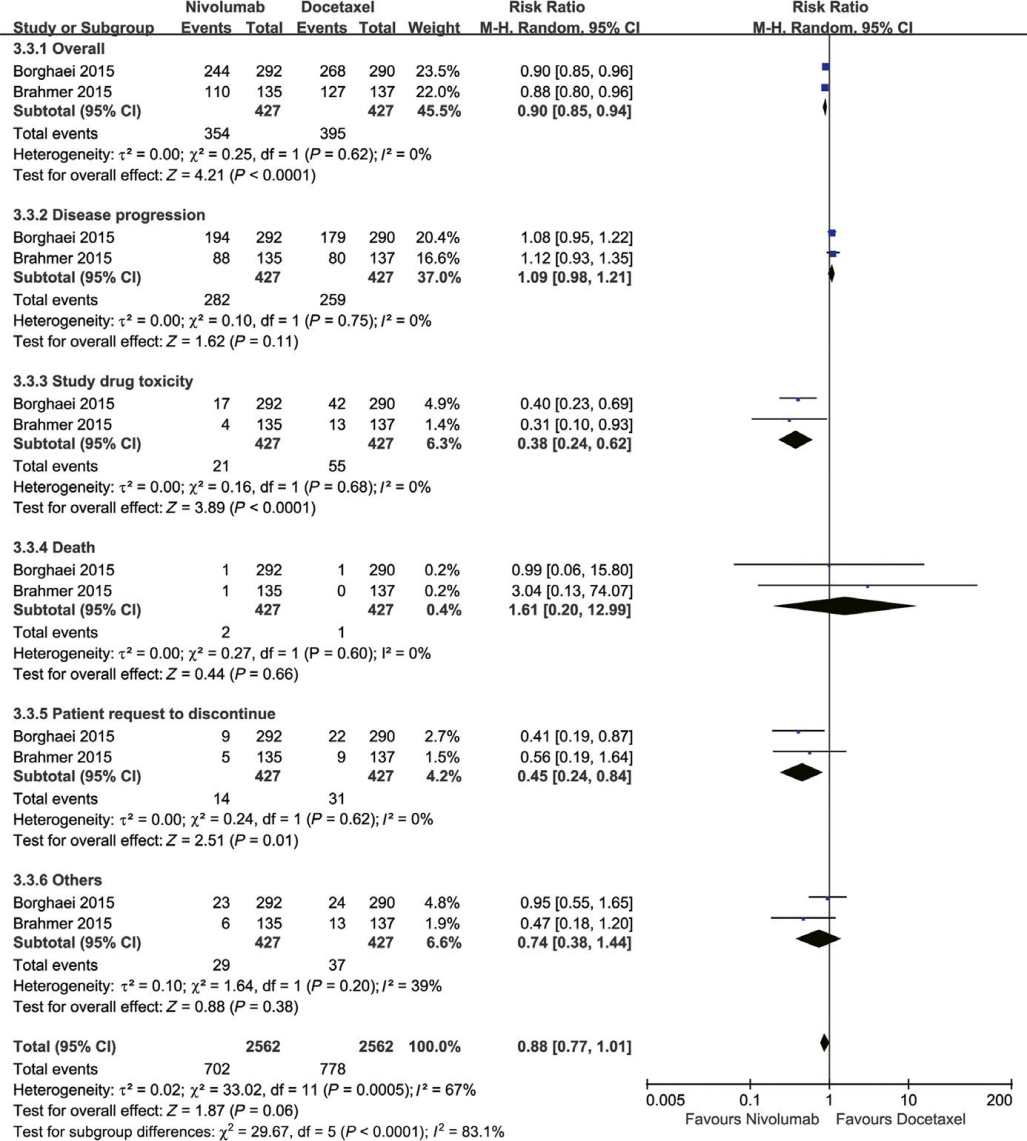
Forest plots of RR of drug discontinuations associated with nivolumab vs docetaxel

More treatment discontinuations were recorded in the docetaxel group (RR: 0.90, 95% CI: 0.85‐0.94, *P* < 0.0001), and this difference was mainly due to drug toxicity (RR: 0.38, 95% CI: 0.23‐0.62, *P* < 0.0001) and the patients’ request to discontinue treatment (RR: 0.45, 95% CI: 0.24‐0.84, *P* = 0.01). The rate of treatment discontinuation due to disease progression was similar in both groups (RR: 1.09, 95% CI: 0.98‐1.21, *P* = 0.10) (Figure 7).

### 3.4 Subgroup analysis

To determine whether the anti‐tumor efficacy of nivolumab compared with that of docetaxel was consistent across various subgroups, the pooled efficacy in terms of PFS and OS was estimated within the various subgroup defined based on the following classification variables: PD‐L1 expression, ECOG status, histology, and study design (Table 3).

**Table 3:** Subgroup analysis for PFS and OS according to PD‐L1 expression, ECOG status, histology, and study design

**Group**	No.of studies	No.of patients		PFS			OS		
		Niv	Doc	HR (95% CI)	*P*	*I^2^* (%)	HR (95% CI)	*P*	*I^2^* (%)
Total	4	469	480	0.70 (0.51‐0.97）	0.03	64	0.70 (0.60‐0.82)	<0.00001	0
PD‐L1 expression									
≥1%	2	186	179	0.69 (0.55‐0.87)	0.002	0	0.66 (0.53‐0.83)	0.0004	0
≥5%	2	137	125	0.54 (0.41‐0.71)	<0.0001	0	0.50 (0.39‐0.64)	<0.00001	0
≥10%	2	122	112	0.54 (0.47‐0.61)	<0.00001	0	0.49 (0.37‐0.63)	<0.00001	0
≥50%	2	83	56	‐	‐	‐	0.42 (0.28‐0.63)	<0.0001	0
<1%	2	162	153	0.90 (0.51‐1.61)	0.73	80	0.74 (0.47‐1.16)	0.19	70
<5%	2	211	207	1.01 (0.58‐1.74)	0.98	82	0.86 (0.60‐1.23)	0.41	58
<10%	2	226	220	0.93 (0.53‐1.64)	0.8	97	0.86 (0.60‐1.21)	0.38	57
ECOG status									
0 or 1	2	427	427	0.77 (0.52‐1.13)	0.17	82	0.71 (0.61‐0.82)	<0.00001	25
Unrestricted	2	42	53	0.53 (0.30‐0.94)	0.03	0	0.59 (0.24‐1.47)	0.26	0
Histology									
Squamous	1	135	137	0.62 (0.47‐0.81)	0.0006	N/A	0.62 (0.47‐0.81)	0.0004	N/A
Nonsquamous	1	292	290	0.92 (0.77‐1.10)	0.37	N/A	1.04 (0.93‐1.15)	0.51	N/A
Unrestricted	2	42	53	0.53 (0.30‐0.94)	0.03	0	0.59 (0.24‐1.47)	0.26	0
Study design									
Retrospective study	2	42	53	0.53 (0.30‐0.94)	0.03	0	0.59 (0.24‐1.47)	0.26	56
RCT	2	427	427	0.77 (0.52‐1.13)	0.17	82	0.71 (0.61‐0.82)	<0.00001	0

Doc, docetaxel; ECOG, Eastern Cooperative Oncology Group; HR, hazard ratios; N/A, not available; Niv, nivolumab; OS, overall survival; PD‐L1, programmed death ligand‐1; PFS, progression‐free survival; RCT, randomized controlled trial.

**Figure 8:**
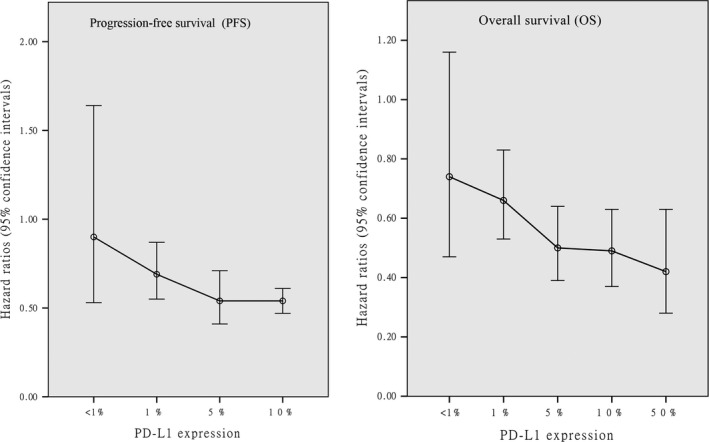
The PFS and OS associated with nivolumab vs docetaxel according to PD‐L1 expression

The subgroup analysis suggested that the anti‐tumor efficacy of nivolumab was superior for squamous NSCLC than for nonsquamous NSCLC in terms of both PFS and OS, and no changes in these endpoints were found among the groups with different ECOG statuses, histological features, and study designs. However, the anti‐tumor efficacy of nivolumab for NSCLC was positively correlated with the level of PD‐L1 expression in terms of both PFS (compared with docetaxel, HR: <1%→≥1%→≥5%→≥10%, 0.90→0.69→0.54→0.54) and OS (compared with docetaxel, HR: <1%→≥1%→≥5%→≥10%→≥50%, 0.74→0.66→0.50→0.49→0.42) (Figures 2, 3, 8, S2 and S3).

### 3.5 Tracking analysis of Checkmate 017 and Checkmate 057

Two included trials^11^, ^12^ presented the 2‐ and 3‐year outcomes from CheckMate 017^10^ and CheckMate 057.^9^ We did not find any significant differences in the anti‐tumor efficacy and toxicity after extension of the study periods (Table 4).

**Table 4:** Tracking analysis of PFS, OS, and Toxicity in Checkmate 017 and Checkmate 057

Study			PFS		OS		Toxicity			
			Median (95% CI) months	HR (95% CI)	Median (95% CI) months	HR (95% CI)	Total AEs %		Grade 3‐5 AEs %	
2015	Borghaei^9^	Checkmate 057	2.3 (2.2‐3.3)	0.92 (0.77‐1.11)	12.2 (9.7‐15.0)	0.73 (0.59‐0.89)	55.5	64.4	10.3	9.1
2015	Brahmer^10^	Checkmate 017	3.5 (2.1‐4.9)	0.62 (0.47‐0.81)	9.2 (7.3‐13.3)	0.59 (0.44‐0.79)	68.1		6.67	
2017	Horn^11^	Checkmate 017	3.5 (2.1‐5.1)	N/A	9.2 (7.3‐12.6)	0.62 (0.47‐0.80)	68		10	
		Checkmate 057	2.3 (2.2‐3.4)	N/A	12.2 (9.7‐15.1)	0.75 (0.63‐0.91)				
2018	Vokes^12^	Pooled Checkmate 017 and Checkmate 057	2.56 (2.2‐3.48)	0.8 (0.69‐0.92)	11.1 (9.2‐13.1)	0.70 (0.61‐0.81)	68		10	

HR, hazard ratios; N/A, not available; OS, overall survival; PFS, progression‐free survival.

### 3.6 Publication bias

There was no evidence of publication bias for PFS (Begg's test *P* = 0.734; Egger's test *P* = 0.272, Figure S4A) and OS (Begg's test *P* = 1.000; Egger's test *P* = 0.640, Figure S4B).

## 4 DISCUSSION

Immune checkpoint inhibitors represent the new standard of care for advanced NSCLC after first‐line treatment. Compared with pembrolizumab and atezolizumab, nivolumab exhibits some advantages in terms of anti‐tumor efficacy and toxicity in the treatment of NSCLC patients.^7^ Docetaxel is considered the standard second‐line treatment for advanced NSCLC and is used as for comparison purposes in this study. This study constitutes the first meta‐analysis to compare nivolumab and docetaxel‐based chemotherapy in advanced NSCLC patients. In summary, the patients that were administered the nivolumab treatment lived longer (both PFS and OS), exhibited better responses and suffered fewer AEs compared with the patients treated with docetaxel. Further analysis indicated that the anti‐tumor efficacy of nivolumab for NSCLC in terms of both the PFS and OS outcomes was positively correlated with the level of PD‐L1 expression. Most of the results from the sensitivity and subgroup analyses were robust.

The main benefit of nivolumab treatment is the significantly longer PFS and OS, as observed in all the included studies, and in CheckMate 063, the anti‐tumor efficacies of nivolumab and docetaxel were similar inthe treatment of NSCLC.^9^, ^10^, ^16^ Together, these trials encouraged the FDA to approve nivolumab as the second‐line treatment for NSCLC after the failure of platinum‐based chemotherapy.^17^ Recent studies have shown that nivolumab provides satisfactory benefits in the treatment of NSCLC. Gettinger et al^18^ suggested that nivolumab treatment could result in increases in long‐term OS (5‐year OS rate: 16%) and durable responses for patients with pretreated advanced NSCLC. In CheckMate 026, nivolumab showed a more favorable safety profile, with similar PFS and OS, for advanced NSCLC than platinum‐based chemotherapy.^19^ Gauvain et al^20^ showed a similar intracerebral activity compared with its reported extracerebral efficacy, with a satisfactory safety profile. Thus, we hypothesize that ICIs, such as nivolumab, are expected to become substitutes for traditional chemotherapy in the near future. Moreover, a subgroup analysis suggested that the anti‐tumor efficacy of nivolumab was superior for squamous NSCLC than for nonsquamous NSCLC in terms of both PFS and OS. Similar results were obtained by Carbone et al^19^ However, all these results are indirect inferences, and more high‐quality RCTs are needed to confirm these findings.

Our meta‐analysis showed that although the nivolumab group exhibited higher ORRs (19.4% vs 11.2%, *P* = 0.0008), it also showed higher rates of disease progression (43.3% vs 31.1%, *P* = 0.0003), which could be due to the following: (a) not all NSCLC issues showed sufficiently high expression of PD‐L1, which plays an important role in the function of nivolumab but does not affect docetaxel‐based chemotherapy, and (b) more or less, docetaxel‐based chemotherapy nearly always exhibited anti‐tumor efficacy for NSCLC. Borghaei et al^9^ suggested that the benefit of using nivolumab, as indicated by all therapeutic endpoints, was greater in patients with PD‐L1‐expressing tumors than in patients without PD‐L1‐expressing tumors. These effects were also demonstrated by Brahmer et al^10^ Thus, we suggest that the examination of PD‐L1 expression is essential before the use of PD‐1 inhibitors (eg, nivolumab). Meanwhile, both Checkmate 017 (not reached vs 8.4 months) and Checkmate 057 (17.2 vs 5.6 months) revealed that the nivolumab group exhibited a significantly longer response time,^9^, ^10^ and both a higher ORR and a longer response time contribute to longer PFS and OS.

PD‐L1 expression was found to be an important indicator in the evaluation of PD1/PD‐L1antibody therapy for NSCLC. The latest NCCN guidelines (2018) suggested a cutoff value for PD‐L1 expression of a least 50% for the use of PD1/PDL1 antibody therapy as the first‐line treatment for NSCLC.^21^ However, different studies have indicated the existence of some controversy regarding the cutoff PD‐L1 expression value for a patient to be considered PD‐L1‐positive.^22^, ^23^ Our meta‐analysis showed a positive correlation for OS and PFS with PD‐L1 expression. Moreover, in the subgroup analysis of the patients with PD‐L1 expression levels of ≥1%, 5%, 10%, and 50%, the OS and PFS rates were significantly elevated by nivolumab treatment compared with treatment with docetaxel. However, a negative correlation was found if the PD‐L1 expression levels were less than 1%, 5%, and 10%. Thus, we suggest 10% as a suitable cutoff value for thePD‐L1 expression level for the treatment of NSCLC patients with nivolumab. More high‐quality RCTs are needed to confirm whether the suggested cutoff PD‐L1 expression value is accurate for nivolumab treatment.

Severe toxicity was a major drawback for traditional docetaxel or platinum‐based chemotherapy and was a common reason for treatment discontinuation. Our meta‐analysis showed fewer incidences of AEs andless severe AEs in the nivolumab group. In comparison with pembrolizumab and atezolizumab, nivolumab also shows benefits in terms of safety.^7^ However, the incidences of immune‐mediated AEs obtained with nivolumab, similarly to those found with pneumonitis and hypothyroidism, remain to be investigated. Scott et al^24^ showed that nearly 30% of NSCLC patients need glucocorticoid treatment during nivolumab therapy, which would decrease the clinical benefits and shorten the OS. Although these immune‐mediated AEs were always of low severity and could be managed based on the established guidelines, they could also be life‐threatening when not given sufficient attention.^25^

The current study had several limitations. First, only six studies (four RCTs and 949 patients) were included, which might affect the reliability of the results, even though the included studies were of high quality. Second, similarly to most other meta‐analyses, we extracted data from published articles without individual patient data, which might increase the heterogeneity between the studies and limit the subgroup analyses. Third, the reported PD‐L1 expression levels were inconsistent between the studies. Although the subgroup analysis of different PD‐L1 expression levels could indirectly indicate a trend, it was unable to show a clear dose‐effect relationship between anti‐tumor efficacy and the PD‐L1 expression levels. Fourth, the types of NSCLC were not identical between the studies, which might have increased the heterogeneity and affected the reliability of the results. Fifth, significant heterogeneity existed in some comparisons, which might have affected the reliability of the results.

## 5 CONCLUSION

Our results suggested that nivolumab is a better choice than docetaxel‐based chemotherapy for advanced NSCLC due to its improved anti‐tumor efficacy (PFS, OS, and ORR) and decreased toxicity. The anti‐tumor efficacy of nivolumab for NSCLC in terms of both PFS and OS showed a positive correlation with the level of PD‐L1 expression. However, due to the inherent limitations of the study, more large‐scale and high‐quality RCTs are needed to support this conclusion. Moreover, the use of a drug combination for lung cancer is also a promising research direction and deserves attention.

## CONFLICT OF INTEREST

All authors declared no conflict of interest to this work.
