# Supplementary figures and images for: Nivolumab provides improved effectiveness and safety compared with docetaxel as a second‐line treatment for advanced non‐small cell lung cancer: A systematic review and meta‐analysis
Source: Cancer Med. 2019 Jan 9;8(2):629–42. doi: 10.1002/cam4.1966 (PMC6382729; doi:10.1002/cam4.1966)

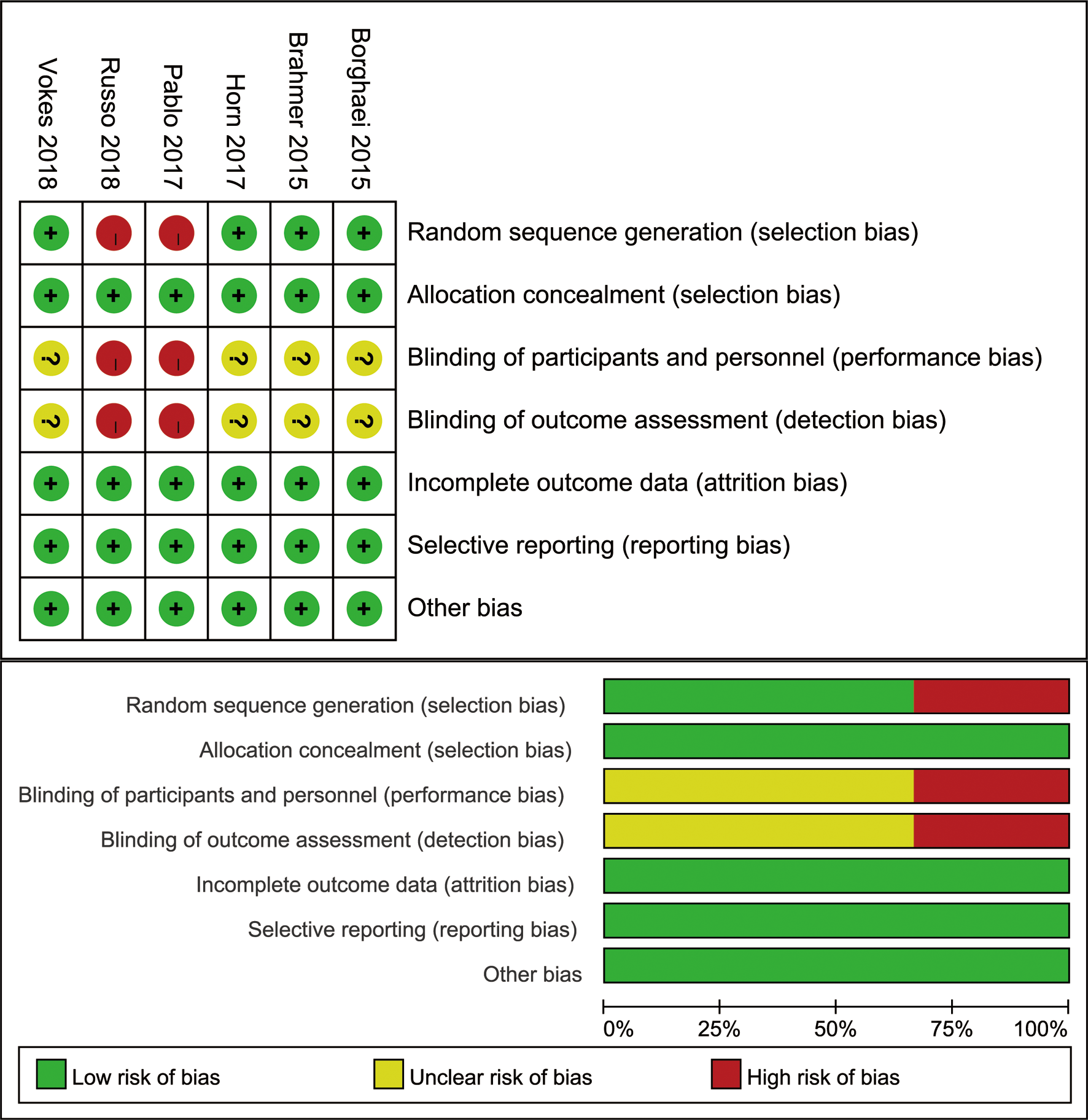

Supplement: Supplementary file 1 [file CAM4-8-629-s001.tif]

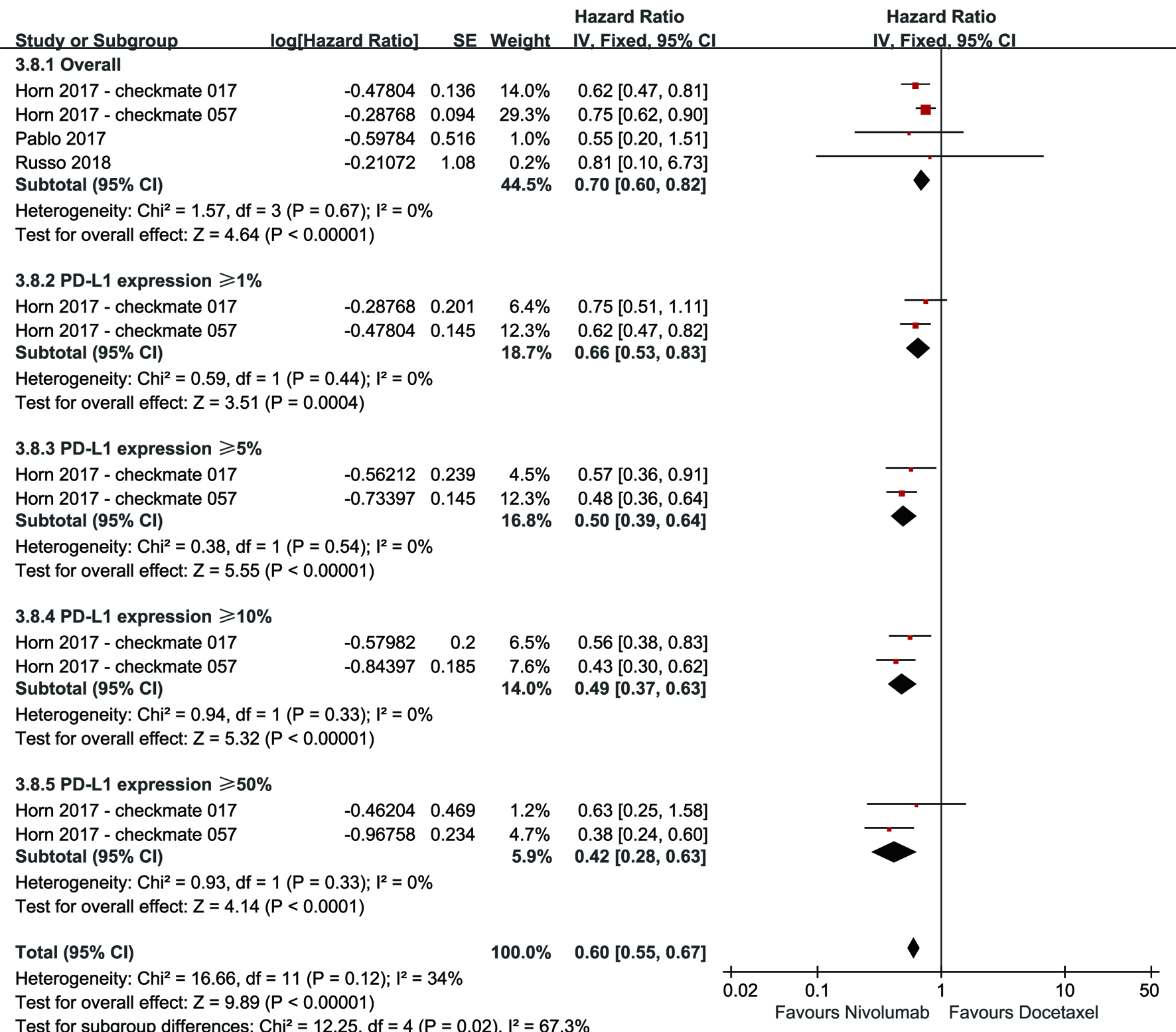

Supplement: Supplementary file 2 [file CAM4-8-629-s002.tif]

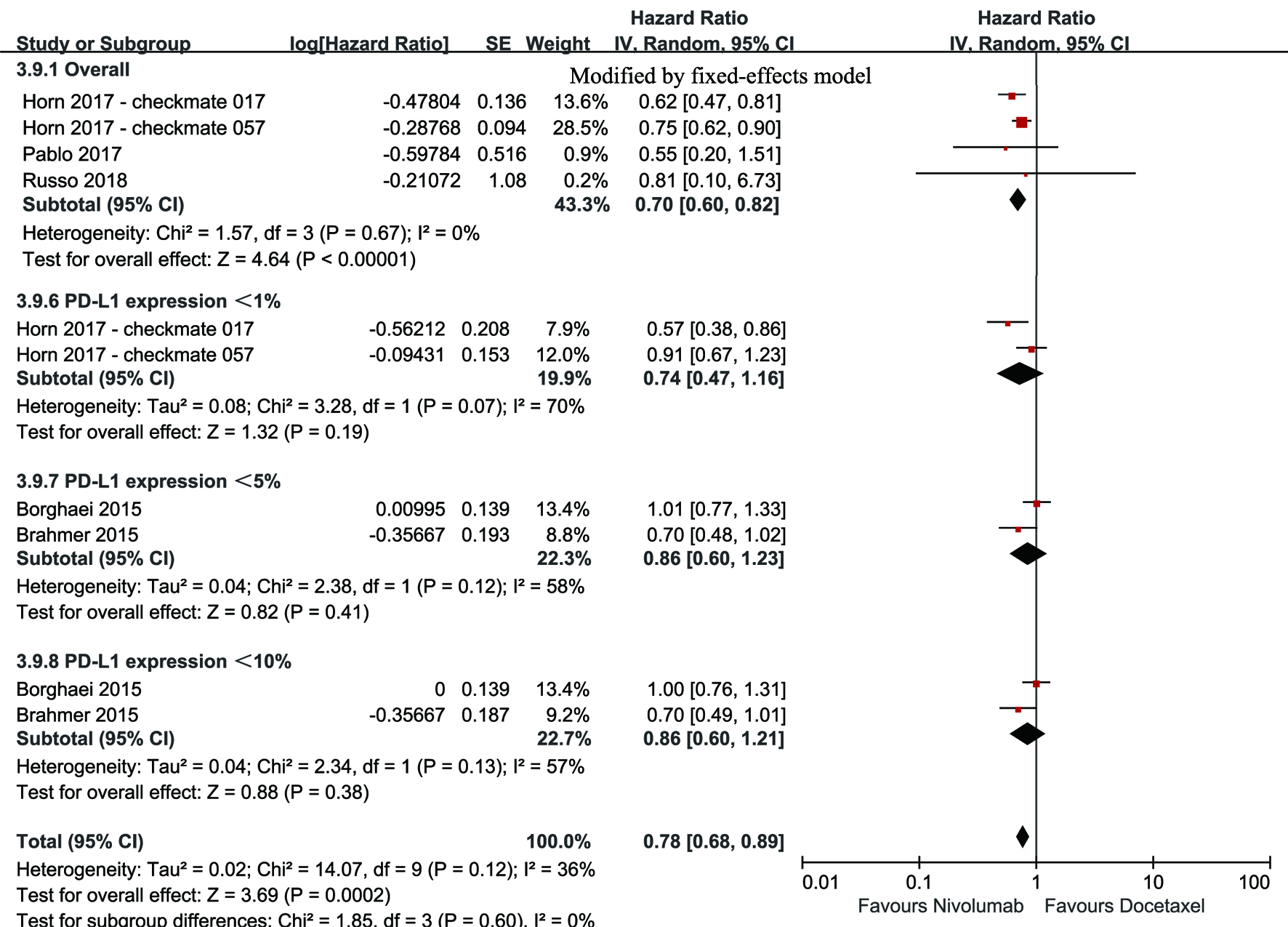

Supplement: Supplementary file 3 [file CAM4-8-629-s003.tif]

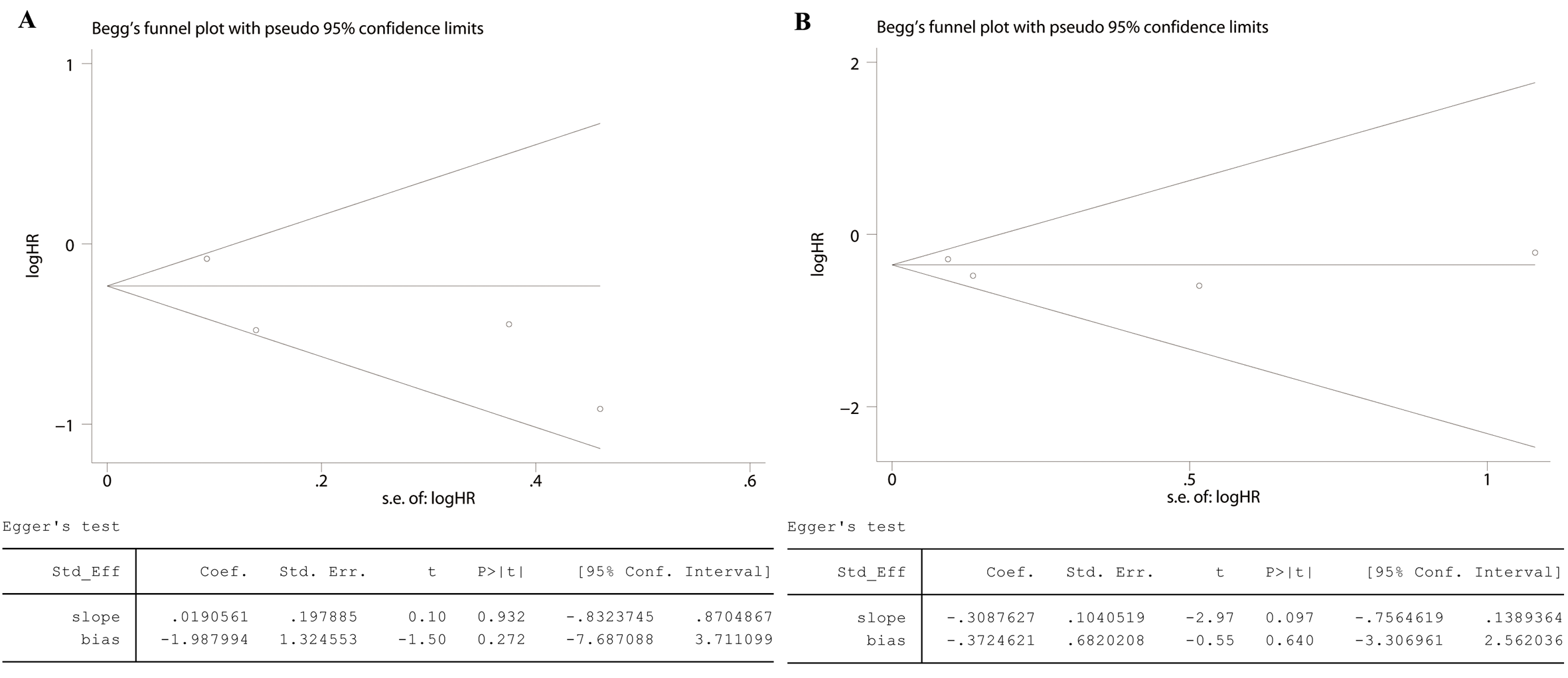

Supplement: Supplementary file 4 [file CAM4-8-629-s004.tif]
